# Virus Diversity and Loads in Crickets Reared for Feed: Implications for Husbandry

**DOI:** 10.3389/fvets.2021.642085

**Published:** 2021-05-20

**Authors:** Joachim R. de Miranda, Fredrik Granberg, Matthew Low, Piero Onorati, Emilia Semberg, Anna Jansson, Åsa Berggren

**Affiliations:** ^1^Department of Ecology, Swedish University of Agricultural Sciences, Uppsala, Sweden; ^2^Department of Biomedical Sciences and Veterinary Public Health, Swedish University of Agricultural Sciences, Uppsala, Sweden; ^3^Department of Anatomy, Physiology and Biochemistry, Swedish University of Agricultural Sciences, Uppsala, Sweden

**Keywords:** metagenome, virome, *Acheta domesticus* densovirus, invertebrate iridovirus 6, *Acheta domesticus*, cricket rearing, frass, *Acheta domesticus* iflavirus

## Abstract

Insects generally have high reproductive rates leading to rapid population growth and high local densities; ideal conditions for disease epidemics. The parasites and diseases that naturally regulate wild insect populations can also impact when these insects are produced commercially, on farms. While insects produced for human or animal consumption are often reared under high density conditions, very little is known about the microbes associated with these insects, particularly those with pathogenic potential. In this study we used both target-free and targeted screening approaches to explore the virome of two cricket species commonly reared for feed and food, *Acheta domesticus* and *Gryllus bimaculatus*. The target-free screening of DNA and RNA from a single *A. domesticus* frass sample revealed that only 1% of the nucleic acid reads belonged to viruses, including known cricket, insect, bacterial and plant pathogens, as well as a diverse selection of novel viruses. The targeted screening revealed relatively high levels of *Acheta domesticus* densovirus, invertebrate iridovirus 6 and a novel iflavirus, as well as low levels of *Acheta domesticus* volvovirus, in insect and frass samples from several retailers. Our findings highlight the value of multiple screening approaches for a comprehensive and robust cricket disease monitoring and management strategy. This will become particularly relevant as-and-when cricket rearing facilities scale up and transform from producing insects for animal feed to producing insects for human consumption.

## Introduction

Most insect species have very high reproductive rates, leading to boom-bust population dynamics regulated by a combination of competition, predation and disease. Disease in particular can have dramatic effects on insect reproduction, growth and survival, at both the individual and population level (1). However, compared to other domestic livestock, very little is known about the prevalence and impact of diseases in commercially reared insects, or about the potential for disease management in high density insect cultivation (2). The notable exceptions are beneficial insects with a long history of commercialization, such as honeybees (3) and silkworms (4, 5). The rapidly emerging insects-as-feed-and-food industry has major knowledge, awareness and research gaps concerning the diseases specific to this industry (6, 7). These are diseases that could significantly impact the production, processing and commercialization of both the insects and their derived products (8). There is consequently an urgent need to develop expertise on insect health and pathology, as the small-scale harvesting of wild insects transitions to commercial insect cultivation, and rearing insects for animal feed develops into rearing for human consumption (7). The feed-food insect rearing industry presently focuses on a few insect species, with Orthopteran insects (grasshoppers and crickets) constituting approximately half the volume of insects reared (9–11). The house cricket (*Acheta domesticus*) and the two-spotted cricket (*Gryllus bimaculatus*) are expected to be important species for the developing insects-as-food market (12). Diseases caused by viruses account for much of the economic impact of the diseases affecting mass-reared insects in historical industries such as apiculture and sericulture (3, 5). This is partly because viruses are particularly well suited to the natural boom-bust population dynamics and high local and temporary densities of many insect species. The vast majority of viruses in the world are asymptomatic (13). Only relatively few viruses are consistently pathogenic, invariably in situations with a high density or continuous supply of susceptible hosts. The risk with viruses lies primarily in their capacity to adapt rapidly to changing circumstances, particularly those governing transmission (13–15). These circumstances are very specific for each individual virus, but the process can be extremely powerful, capable of quickly transforming an insignificant, asymptomatic virus into a major pathogen (16). These are important considerations for the nascent insect feed-food industries, which need to produce large numbers of healthy individuals in the least amount of time and space, i.e., ideal criteria for virus transmission, disease and virulence evolution. This is a reason why viruses have been particularly singled out as a potential threat to the industry (17). Unfortunately, our knowledge of insect viruses is heavily skewed toward those insects that are damaging to human progress, either as vectors of viral diseases to humans or their domesticated plants and animals, or as biocontrol agents of insect pests (13). Very little is known about viruses of insects in general, although major efforts have been made recently to at least catalog the virus diversity in a wide range of insect and invertebrate species (18–26). Orthopteran insects in particular are underrepresented in virological research, despite the pressing need for screening, quarantine and disease management protocols for rearing Orthopteran insects to acceptable animal welfare and food-feed health and safety standards. This study takes a first step toward redressing these imbalances, by characterizing novel and known viruses in commercially reared *A. domesticus* and *G. bimaculatus*. For this we used two complementary approaches: a target-free exploration of cricket frass nucleic acid, for discovering new potential virus hazards, and a targeted screening of a limited set of insect and frass samples from several local Swedish cricket retailers and producers, for detecting and quantifying known viral pathogens of crickets. This targeted screening focused on nine different viruses, of which seven (*Acheta domesticus* densovirus - AdDV, invertebrate iridovirus 6 - IIV-6, *Gryllus bimaculatus* nudivirus - GbNV, *Acheta domesticus* volvovirus - AdVVV, *Acheta domesticus* mini ambidensovirus - AdMADV, *Acheta domesticus* virus - AdV and cricket paralysis dicistrovirus - CrPV) have been previously associated with the rearing of crickets (27–35). The remaining two viruses are slow bee paralysis virus [SBPV, (36)], which was present at high levels in the target-free nucleic acid exploration of the current study, and a novel Iflavirus recently characterized from wild *A. domesticus* (37).

## Methods

### Sample Collection and Processing

Samples of commercially reared house crickets (*A. domesticus*) and two-spotted crickets (*G. bimaculatus*) were obtained from six different Swedish retailers (identified anonymously by the letters A–F; Supplementary Table 1). The samples were shipped according to the retailers' specifications. Both insects and frass were screened for viruses, since most cricket viruses are acquired orally and shed as particles into the gut lumen (8). Insect homogenates were prepared by pulverizing flash-frozen insects in BioReba meshbags (Bioreba, Reinach, Switzerland) with a pestle and resuspending in 2 mL sterile water per insect. Frass homogenates were prepared in 0.5 mL sterile water per 0.1 g frass using a MixerMill 400 beadmill (Retsch Haan, Germany) and ten 3 mm glass beads shaking at maximum speed for 60 s (38). DNA was extracted from 100 μL homogenate using the Qiagen Blood and Tissue kit (Qiagen, Hilden, Germany) following the “Tissues and Rodent tails” protocol and eluting the DNA in 200 μL AE buffer. RNA was extracted from a separate 100 μL aliquot of homogenate using the Qiagen RNeasy Plant Mini kit, following the “Plant” protocol and eluting the RNA in 50 μL RNase Free water. The DNA/RNA concentration was estimated using a NanoDrop 1,000 instrument (NanoDrop, USA), after which the samples were stored at −20°C until further use.

### Target-Free Exploration - Sequencing and Bioinformatic Analyses

The target-free virus prospecting study was based on bioinformatic analysis of mass parallel sequencing data of nucleic acid (RNA and DNA) extracted from a single frass sample from commercially reared A. *domesticus*. We chose frass for the target-free screening, since most of the cricket viruses associated with commercial rearing are acquired orally and accumulate in the gut lumen until voided into the environment as part of the frass (8). This means that frass nucleic acid will have a higher proportion of viral genomes than nucleic acids from insect tissues, improving the chances of discovering new, low-abundance viruses. Moreover, the viral nucleic acids in frass will be mostly derived from virus particles shed into the gut lumen, while viral nucleic acids from insect tissues will mostly represent replication or transcription-translation intermediates, rather than infectious units. Around 1.5 μg total RNA was depleted of ribosomal RNA using the Illumina RiboZero rRNA depletion kit and submitted for Ion Proton S5XL sequencing while around 1.0 μg of total DNA was submitted for PacBio sequencing, both conducted by LifeSciLab in Uppsala Sweden. The RNA reads were converted to FASTQ format using the SamToFastq tool (39). The DNA reads were delivered in FASTQ format as circular consensus sequencing (CCS) reads. Both the RNA and DNA reads were trimmed and checked for quality control using FastQC (40) and the Fastx-Toolkit (41). The reads were compared against a local copy of the NCBI nr database (downloaded on 3 June 2020) and assigned to a taxonomic group using DIAMOND BLASTx v0.9.31 (42). The quantitative and phylogenetic distributions of the reads were visualized using hierarchical pie charts produced with Krona Tools v2.7 (43). The taxonomic data were evaluated for potential viral pathogens and candidate reference genomes were identified and retrieved from GenBank in FASTA format. A more detailed description of the sequencing and bioinformatic analyses can be found in the Supplementary Material.

### Targeted Screening - PCR-Based Virus Detection and Quantification

The presence and amount of the nine viruses of interest was determined by quantitative PCR in a limited selection of insect and frass samples from commercially reared *A. domesticus* and *G. bimaculatus* (Supplementary Table 1). The viruses with a DNA genome (AdDV, IIV-6, GbNV, AdVVV, AdMADV) were assayed directly from about 5–70 ng of frass/cricket DNA template. For the viruses with RNA genomes (CrPV, AdV, AdIV and SBPV), between 60 and 1,800 ng RNA was first converted to cDNA using the InVitrogen SuperScript-III 1st-strand cDNA kits (ThermoFisher Scientific, Waltham, MA, USA) and diluted 5-fold in ultrapure water. The cDNA equivalent of 1–36 ng original RNA was then used as template for qPCR. The forward and reverse primers for each assay were either obtained from the literature or designed *de novo* (Supplementary Table 2). All primers and assays are compatible with the thermocycling profile for the AdDV VP gene (38). All assays were run in duplicate using the SsoFast EvaGreen Supermix kits (BioRad, Hercules, CA, USA) with the mean Cq value used for quantitative analyses (44). Only data from the first 35 cycles were used for making detection assessments and quantification, due to the risk of false positive or false negative results beyond 35 cycles of amplification (45). The identity of all amplicons of the expected size (Supplementary Table 2) was confirmed by Sanger sequencing (Macrogen Europe, Amsterdam, The Netherlands).

### Statistical Analyses

To compare virus titres across samples and viral burden between the *A. domesticus* suppliers (all except supplier “C”) we created a series of simple Bayesian linear models (Supplementary Appendix 1) to: (1) estimate the viral burden for individuals from each supplier, for each of the five viruses where we detected virus from more than one supplier (AdDV, IIV-6, AdVVV, GbNV and AdIV), and (2) whether the titres of the different viruses were correlated across suppliers.

We used Bayesian models because: (1) the very limited sample sizes preclude any statistical approach other than Bayesian methods, which fit the data to a likelihood function and thus can produce probability estimates from any sized data set, (2) the estimated parameter ranges make it relatively easy to compare the 95% Confidence Intervals of the group-level effects (i.e., comparing estimated viral loads from suppliers) in addition to estimating the probability that the beta parameter >0, which represents correlation between viral titres, and (3) because our hierarchical models use a single distribution to estimate the range of intercepts, this allows parameter estimation for suppliers with little information to “borrow strength” from the entire dataset to maximize the information available and limit the effect of unusually large or small data points when little data exist. A detailed description of the models, equations and statistical packages used can be found in Supplementary Appendix 1.

## Results

### Target-Free Exploration of the *Acheta domesticus* Frass Virome

**Figure 1:**
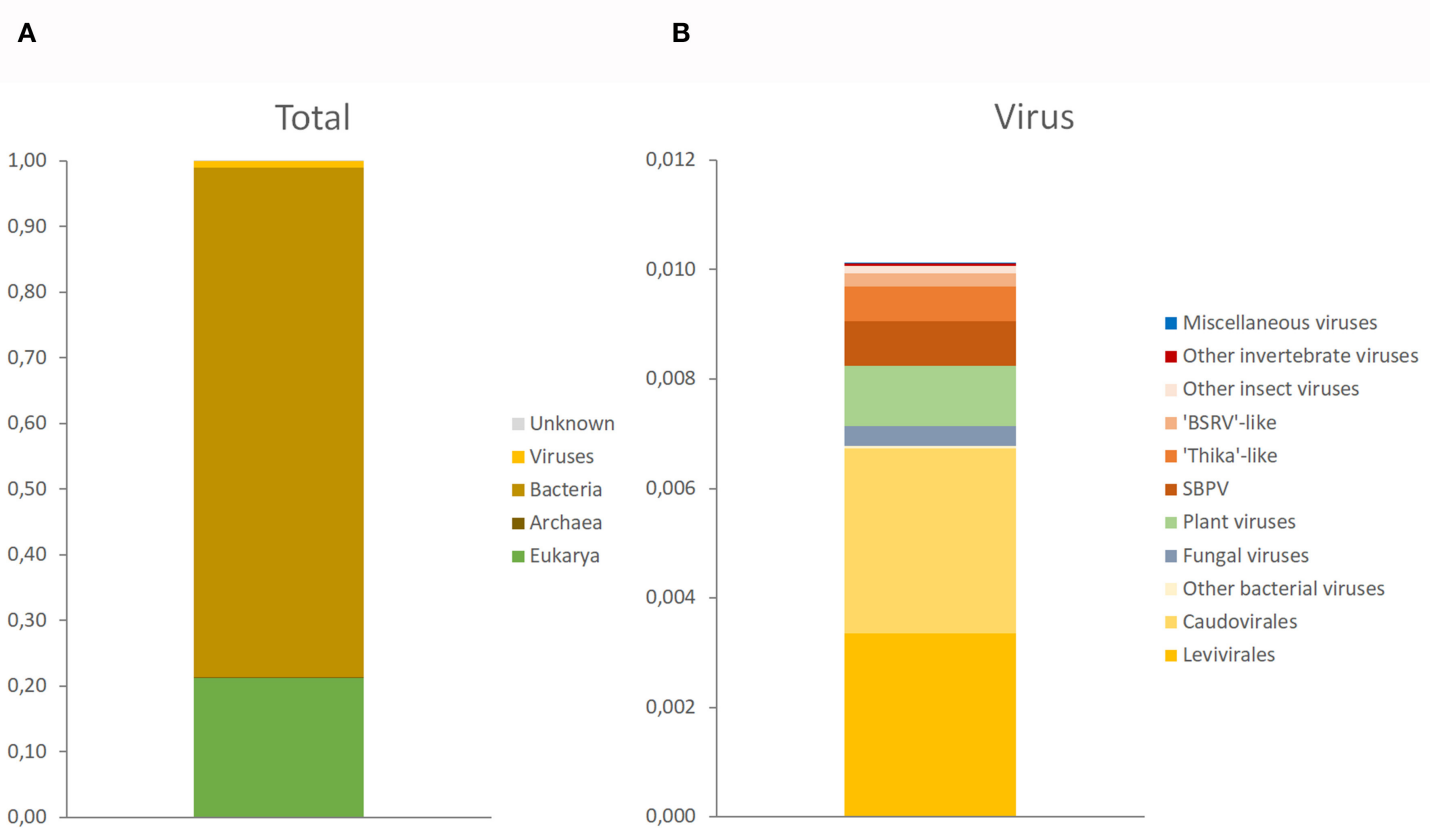
Taxonomic composition of *Acheta domesticus* frass. **(A)** Full composition at the level of domain (Archaea, Bacteria, Eukarya, and Viruses). **(B)** Composition of the 1% virus fraction, separated by primary host: bacteria (yellow), fungi (gray), plants (green), invertebrates (orange-red), and miscellaneous viruses (blue) whose host status is unclear.

**Figure 2:**
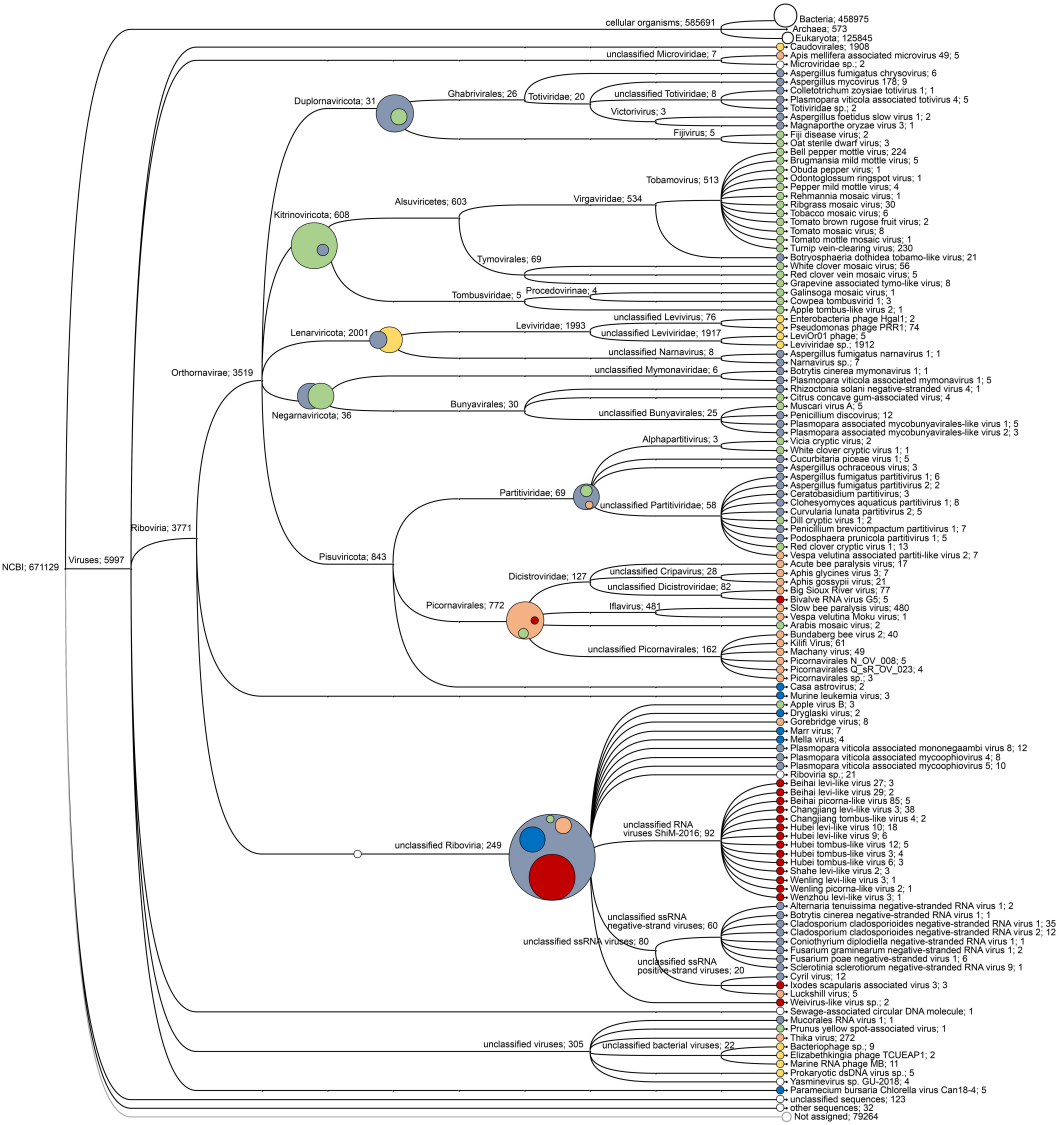
Virus read cladogram and hosts. Cladogram of the viral reads obtained through from the RNA sequencing. The primary hosts of the viruses are indicated by the same colors as for Figure 1: bacteria (yellow), fungi (gray), plants (green), invertebrates (orange-red), and miscellaneous viruses (blue). The composite circles represent the host distribution at higher virus taxonomic categories.

The target-free exploration of *A. domesticus* frass DNA and RNA showed that this frass sample consisted mostly of bacterial and plant material, with viruses comprising a very small fraction (about 1%) of the RNA metagenome and even less of the DNA metagenome (Supplementary Figure 1). The only viruses identified through DNA sequencing were invertebrate iridovirus 6 (IIV-6) and bacteriophages from the Order Caudovirales. The RNA sequencing identified a much larger diversity of viruses, from insects, their microbiome and their (plant) food (Figures 1, 2). Most prominent among these are RNA transcripts of the bacteriophages that were also found in the DNA sequencing, bacteria-infecting RNA viruses from the Order Levivirales, slow bee paralysis virus, an Iflavirus with bumblebees as its suspected primary host (46), a “Thika-like” virus similar to a trio of closely related Drosophila-infecting viruses (Thika virus, Kilifi virus and Machany virus) in a small unassigned clade in the Order Picornavirales (24, 25), and a “BSRV-like” dicistrovirus similar to Big Sioux River virus (47), Bundaberg bee virus 2 (19) and *Aphis gossypii* virus (48). The SBPV reads were >98% identical to the SBPV reference genome sequence (36). Not enough reads were available for the “Thika-like” and “BSRV-like” viruses for detailed genetic characterization or for designing reliable diagnostic assays. In addition to these three groups of relatively abundant reads corresponding to three individual viruses there was also a large group of reads matching a diverse assortment of viruses from a wide range of insects and invertebrates (18), with each virus accounting for just a handful of reads. The next largest category consisted of reads matching viruses that primarily infect plants, mostly belonging to turnip vein-clearing virus and bell pepper mottle virus, 2-well-characterized Tobamoviruses from the Family Virgaviridae (49). Another major group of reads matched a wide range of fungus-infecting viruses, mostly associated with *Cladosporium cladosporioides* (a common mold), *Botryosphaeria dothidea* (a wide-ranging canker-causing plant pathogen) and *Plasmopara viticola* (causative agent of grapevine downy mildew). Finally there were a few reads matching viruses associated with Antarctic penguins and their ticks (26). These data were obtained from just a single frass sample, and can therefore only be used in a descriptive sense, as a case study.

### Targeted Screening of Nine Viruses of Interest

**Figure 3:**
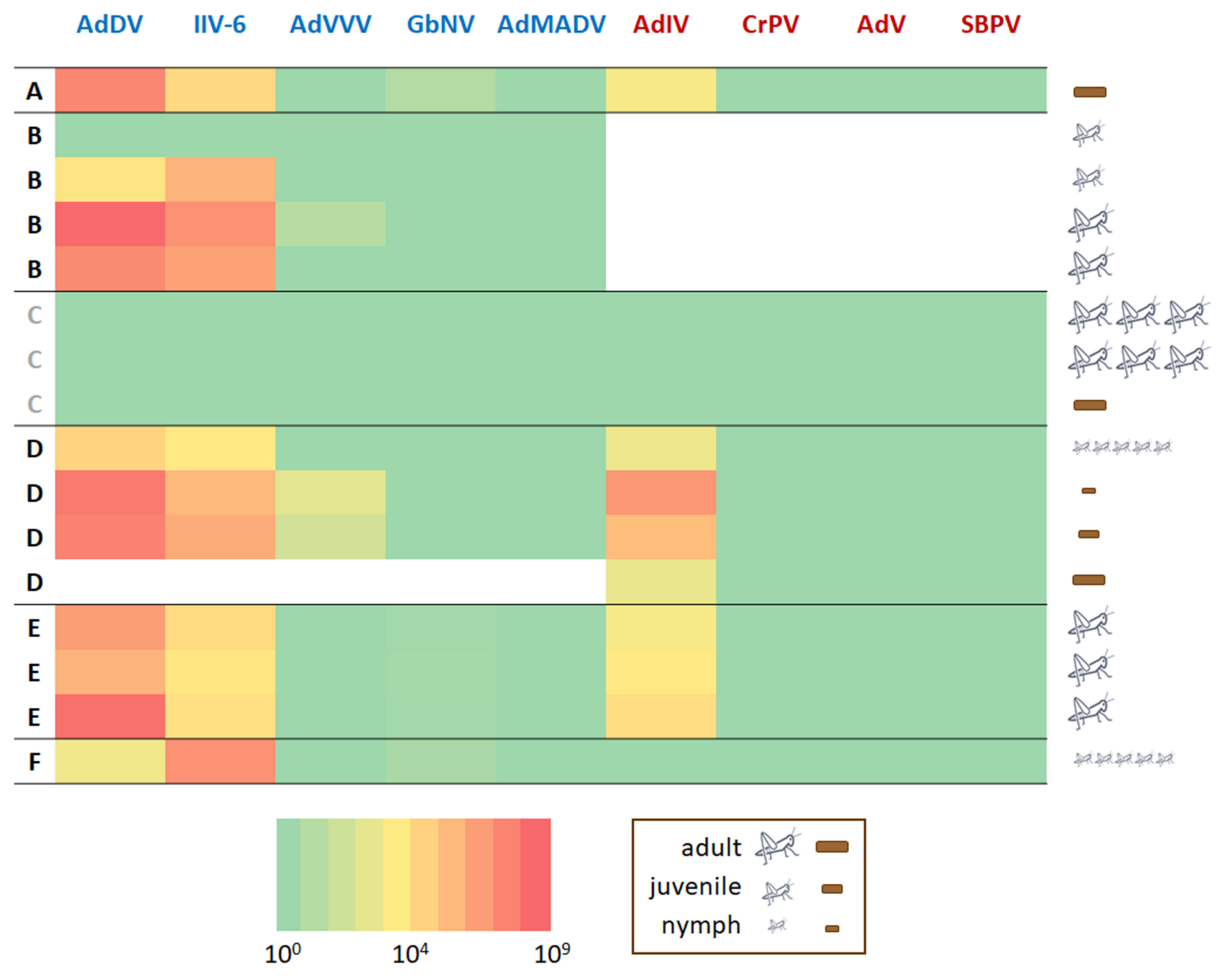
Virus detection & quantification. Heat map of the levels of six viruses in cricket and frass samples from different retailers (A–F). Retailer C (gray font) supplied *Gryllus bimaculatus* crickets. All other retailers (black font) supplied *A. domesticus* crickets. The viruses with a DNA genome: *Acheta domesticus* densovirus (AdDV), Invertebrate iridovirus 6 (IIV-6), *Acheta domesticus* vovovirus (AdVVV), *Gryllus bimaculatus* nudivirus (GbNV), and *Acheta domesticus* mini ambidensovirus (AdMADV) are shown in blue font. The viruses with an RNA genome: *Acheta domesticus* iflavirus (AdIV), Cricket paralysis dicistrovirus (CrPV), *Acheta domesticus* virus (AdV), and slow bee paralysis virus (SBPV) are shown in red font. The legend next to the heat map indicates which sample type (crickets or frass) was analyzed, the developmental stage (adult, juvenile, nymph) the insect or frass sample came from and how many individuals were included in each insect sample (not applicable for frass samples). The heat map is on logarithmic scale and refers to the number of estimated copies of virus detected in the (RT)-qPCR reaction. White fields indicate missing data.

Of the seven viruses previously associated with cricket cultivation, AdDV and IIV-6 were relatively common and abundant in this limited survey of Swedish cultivated crickets. AdVVV and GbNV were detected at relatively low levels in just a few samples, while neither AdMADV, CrPV nor AdV were detected in any of the samples (Figure 3). Slow bee paralysis virus, which was highly abundant in the target-free exploration screen, was not detected in any of the Swedish cultivated cricket samples by targeted screening, while the novel Iflavirus recently identified in wild *A. domesticus* (37) was both very common and abundant in these samples. None of the viruses were detected in the *G. bimaculatus* cricket or frass samples from supplier “C.” Older crickets and their frass tended to have higher levels of AdDV and IIV-6, although there was much variation between individual crickets, while no such tendency was seen for AdIV. There was no significant difference between the five *A. domesticus* suppliers in virus load for AdDV, IIV-6, AdVVV and AdIV (Supplementary Figure 2). The suppliers appeared to differ in GbNV load (Supplementary Figure 2), although this result would need confirmation with larger sample sizes. There was some evidence of correlation between several of the viral titres across samples. AdDV appeared to be positively correlated with both IIV-6 (beta estimate = 0.99 ± 0.30; with 99.3% probability of the beta estimate >0 based on the Bayesian posterior distribution), AdIV (beta estimate = 0.83 ± 0.39; posterior probability of 97.4%) and AdVVV (beta estimate = 1.27 ± 1.0; posterior probability of 91.5%). There was no evidence of correlation with GbNV. Both the analysis of virus loads between suppliers and the correlation between virus titres across samples should be interpreted with caution because of the extreme paucity of data used to inform the models.

## Discussion

Our study revealed that viruses comprised only a very small fraction, around 1%, of the total frass nucleic acid content which was dominated by nucleic acids from bacteria and plants. This is not surprising for a frass sample, which can be expected to consist mostly of intestinal bacteria and the remnants of the food consumed by the crickets. This also applies for the viruses identified in the frass sample, which are linked to either the host, its intestinal bacteria, or the plants consumed. Although the target-free screening approach is comprehensive and ostensibly neutral, its output is contingent on the quality of the genetic databases. These are strongly biased toward organisms with economic significance for human progress, such as our domesticated life forms (e.g., crop plants, production animals (cattle, pigs, poultry, honeybees) and companion animals) and their diseases. Microbial diversity in particular is underrepresented in these databases, and skewed toward pathogenic organisms. This affects both the proportion of reads that can be identified and the type of identification made. The target-free screening in our study identified both specific virus and viruses that could be identified at higher taxonomic levels but not at species level, i.e., novel viruses. The fully identified insect-infecting viruses included IIV-6 (in the DNA fraction) and SBPV (in the RNA fraction). The IIV-6 reads matched the lizard strain of IIV-6, which infects both Orthopteran and reptilian hosts (28), and thus represents a direct potential health hazard for reptilian pets. The SBPV reads were closely related, though not identical, to the SBPV type strain (36). SBPV belongs to the Iflaviridae: an exclusively insect-infecting virus family with a single-stranded RNA genome (50), and is so far primarily found in bumblebees (36, 46). We also identified several viruses of the cricket intestinal microbiome, such as bacteriophages (DNA fraction) and Leviviruses (RNA fraction).

The novel viruses identified at higher taxonomic levels included several moderately abundant viruses from insect-specific virus families in the Picornavirales: a very diverse, widespread and successful order of viruses with hosts throughout the plant and animal kingdoms. These virus families include many pathogenic viruses, which makes these new viruses particularly interesting from a cricket health and disease management perspective. However, there were also some unexpected absences from the target-free exploration. The most obvious of these is the absence of AdDV from the frass DNA fraction, despite the high incidence and abundance of AdDV in similar *A. domesticus* samples revealed by the targeted screening. This discrepancy may have a technical origin. PacBio sequencing requires double-stranded DNA. Iridoviruses and bacteriophages have double-stranded DNA genomes and were therefore readily identified by the PacBio screening. However, densoviruses have single-stranded DNA genomes, with positive and negative ssDNA genome copies packaged in separate particles. Even though these ssDNA genomes can hybridize upon extraction and purification (51), the GC-rich terminal palindromic sequences may make these reconstructed dsDNA genomes inaccessible for PacBio sequencing. Other anomalies, and insights, are revealed by comparing the viral composition of the DNA and RNA fractions. Viruses with RNA genomes [i.e., the vast majority; (18, 19)] will obviously only be recovered in the RNA fraction. However, all viruses with DNA genomes need to transcribe their genes into mRNA prior to translation. They could therefore in theory also be identified in the RNA fraction, and thus provide evidence of both their existence and their replication. This was clearly the case for the bacteriophages, whose sequences were in both the DNA and RNA fractions. Since bacteriophages infect the bacteria of the gut microbiome, this is entirely logical and expected. However, IIV-6 was only identified in the DNA fraction of the frass sample, not in the corresponding RNA fraction. The inference is therefore that only Iridovirus particles were present in the frass, and not any RNA traces of their replication in insect tissues, such as the gut epithelial cells. The same may well also apply to the other DNA viruses detected in similar samples by the targeted screening, such as AdDV, AdVVV, and GbNV.

Comparing the results from the target-free exploration with those of the targeted screening can also be informative. A good example is provided by the target-free and targeted screening results for SBPV and AdIV. In the case of AdIV, the results of the target-free exploration were confirmed by the targeted screening of similar additional samples, clearly establishing this virus as a consistent and abundant part of the *A. domesticus* virome (37). By contrast, SBPV was only identified by target-free sequencing of a single frass sample, and not by the targeted screening of the other insect and frass samples. There are multiple possible explanations for this discrepancy. It is of course entirely possible that the results are real, and that the discrepancy is simply a stochastic consequence of the small sample sizes of the target-free (*n* = 1) and targeted (*n* = 12) screening. Biologically it is certainly not out of the question that an Iflavirus like SBPV could, possibly, be infectious to crickets as well as to bees: this is a simple question of host range and Iflaviruses are very common in all insects that have been screened (18, 19, 50). However, it is also possible that this result is an artifact of the target-free screening workflow. The sheer number of SBPV reads recovered [480] and their even distribution across the SBPV genome argue against either technical or bioinformatic contamination during the sequencing and analysis workflows (52, 53). Although laboratory contamination during RNA extraction cannot be entirely ruled out, it is difficult to envision where and how this could entered the workflow in the amounts required to return the results from the RNA sequencing. Finally, the absence of reads matching other common bee viruses, or bees themselves, argue against passive acquisition of SBPV through feeding commercially reared crickets on contaminated material, e.g., dead bees, bee-collected pollen (54) or plant material contaminated with bee feces (55). In summary, the data presented here is too limited and uncertain to make active determination on the possible status of SBPV as a cricket-infecting virus, which must therefore remain “unproven” until more convincing evidence is obtained.

These insights and logical deductions highlight the value of complementary screening strategies and sample types for a robust holistic assessment of viruses and their potential risks for cricket rearing. The advantage of screening frass samples is that this allows an assessment of the health of the cricket microbiome, such as viral diseases of beneficial bacteria and fungi, as well as the health of the cricket itself. Since the main functions of the microbiome are in food metabolism and protection against diseases (56), the health of the microbiome is directly relevant to the health of the cricket, and thus also relevant to cricket husbandry and health management. Targeted screening is very precise and accurate but only detects what it is being asked to detect. It is therefore useful for monitoring known threats but not for explaining new diseases or identifying potential future threats. A health strategy based exclusively on targeted screening for known pathogenic agents therefore brings a risk of potential misdiagnosis, and consequently an inappropriate management strategy. These strengths and weaknesses are reversed for target-free screening, which can identify all viruses present in a particular sample but not distinguish between those that are benign and those that are pathogenic. It is therefore very good at identifying potential threats and new disease associations, but is less accurate for monitoring actual threats. It is also still subject to a number of workflow and bioinformatic errors, uncertainties and biases, as we also discovered in this study. However, a combination of the two screening approaches maximizes their individual strengths, minimizes their respective weaknesses, and reduces the risk of misinformation and mismanagement, to form a solid basis for a robust disease monitoring and management strategy.

Viruses are a natural part of life. The diversity of viruses detected in these particular cricket samples is, in and of itself, neither unusual nor alarming. The new viruses identified add to the growing list of novel viruses identified from insects and invertebrates, and likely represents only a fraction of the complete cricket virome. It is impossible to predict *a priori* which of these will develop into a biohazard for the cricket industry: that depends on the compatibility of the cricket rearing conditions with the transmission characteristics of each virus and can only be determined experimentally.

However, the frequent detection of high titres of several known pathogenic cricket viruses in just a small selection of commercially sourced orthopterans is alarming. It demonstrates that these pathogenic viruses are probably widespread in the local cricket rearing and retailing facilities. This is especially concerning in the light of the minimal regulation or sanitary control in the extensive trade and movement of Orthopertans (1, 27). These viruses are known to significantly impact both individual and population health, and represent a direct major biological and economic risk for the cricket rearing industry (8, 17, 27, 57). The retailers sampled in this study sold crickets as feed for pets, such as reptiles, amphibians and spiders. The cricket rearing criteria for this market are less stringent that those for rearing crickets for human consumption, whose operations may therefore be less affected. Both the silkworm and bumblebee rearing industries have significantly reduced their overall disease profile and risk for epidemic spread through high hygienic standards, containment measures, regular monitoring and tight control over external inputs into their operations. The current practices for rearing, movement and sale of crickets for feed and food are not yet up to these standards (58, 59), so our findings are likely of interest for a large part of the rearing sector (60, 61).

In the meantime, the following recommendations can help limit the spread and potential impact of viruses, and other diseases:

(1) Only incorporate new individuals into a population after testing for relevant pathogens
(2) Quarantine individuals before release into the population
(3) Monitor regularly the pathogen status of the population
(4) Quarantine with any change in mortality, reproduction or behavior of animals
(5) Keep informed on new developments concerning the health and pathology of the insect species reared.

## Data Availability Statement

The sequence data analyzed in this study have been deposited in the GenBank small read archives, under accession numbers SRR13582031 (DNA) and SRR13582032 (RNA), Study accession SRP303879, Bioproject accession PRJNA697972 and Biosample accession SAMN7703531.

## Author Contributions

ÅB, AJ, and JMd conceived and designed experiments. ES, PO, and FG performed experiments. FG, ML, JdM, and ES analyzed the data. AJ and ÅB contributed reagents, materials, and analysis tools. JdM and ÅB wrote the initial manuscript. All authors contributed to the article and approved the submitted version.

## Conflict of Interest

The authors declare that the research was conducted in the absence of any commercial or financial relationships that could be construed as a potential conflict of interest.
