# Supplementary material for: Virus Diversity and Loads in Crickets Reared for Feed: Implications for Husbandry
Source: Front Vet Sci. 2021 May 20;8:642085. doi: 10.3389/fvets.2021.642085 (PMC8173086; doi:10.3389/fvets.2021.642085)
Supplement: Supplementary file 1 [file Data_Sheet_1.PDF]

Supplementary Material

1. Supplementary Data

Around 1,5 μg of total RNA extracted from a single frass sample from commercially reared *A. domesticus* was submitted for Ion Proton S5XL sequencing by LifeSciLab in Uppsala Sweden. Ribosomal RNA was removed from the sample using the Illumina RiboZero rRNA depletion kit. The remaining RNA was quantified and checked for integrity using the Agilent Bioanalyzer and the sequencing library was constructed using the Ion Total RNA-Seq v2 kit. The library was barcoded and sequenced on an Ion S5XL instrument in an IonChef S5 530 chip, together with similarly barcoded RNA sequencing libraries from bumblebees and Indian tasar silkworms. Around 1,0 μg DNA from the same frass sample was also submitted for PacBio sequencing by LifeSciLab in Uppsala Sweden, in a single barcoded sequencing run together with honeybee DNA samples. The Ion S5XL reads were delivered demultiplexed in BAM files and converted to FASTQ format using the SamToFastq tool in the Picard package v2.23.4 (Picard Toolkit, 2019). The PacBio-reads were demultiplexed and delivered as circular consensus sequencing (CCS) reads in FASTQ format. Both the Ion S5XL and PacBio reads were trimmed and passed through quality control using FastQC (Andrews 2010) and the Fastx-Toolkit (Gordon and Hannon 2010). Taxonomic assignment was performed by comparing reads against a local copy of the NCBI nr database (downloaded on 3 June 2020) using DIAMOND BLASTx v0.9.31 (Buchfink *et al.* 2015). The resulting DAA files were imported into MEGAN6 v 6.19.7 (Huson *et al.* 2016) using the following mapping file: megan-map-Jul2020-2.db. The quantitative and phylogenetic distributions were also visualized using hierarchical pie charts produced with Krona Tools v2.7 (Ondov *et al.* 2011). The taxonomic data were evaluated for potential viral pathogens and candidate reference genomes were identified and retrieved from GenBank in FASTA format. Assembly of reads was performed using both SPAdes v3.11.1 (Bankevich *et al.* 2012) and MegaHit v1.1.2 (Li *et al.* 2015) with default settings. Contigs were imported into CodonCode Aligner v8.0.1 (CodonCode Corporation, Dedham, MA, USA) and Geneious Prime v2020.2.4 (Biomatters Ltd., Auckland, New Zealand; Kearse *et al.* 2012) in order to assess quality, merge partially overlapping contigs, and compare with candidate reference genomes.

2. Supplementary Figures and Tables

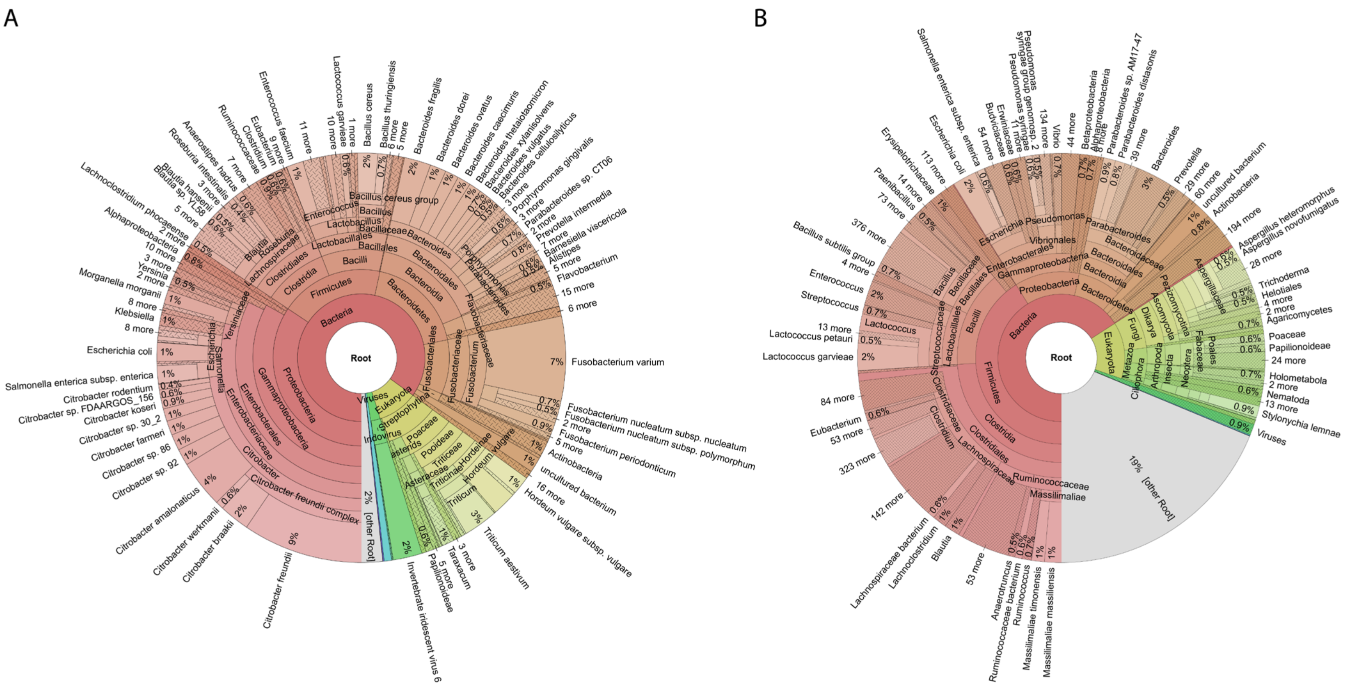

Supplementary Figure 1. Taxonomic composition of the *A. domesticus* frass sample as determined from target-free sequencing analysis of the frass DNA (A) and RNA (B) samples. About two-fifths of the bacterial reads in both the DNA and RNA sequencing were assigned to the Phylum *Proteobacteria*: 75% to a variety of *Citrobacter* species with the remaining 25% assigned to a diverse range of *Enterobacterales*, including a number of well-known pathogenic taxa, such as *Salmonella enterica*, *Escherichia coli*, *Klebsiella spp.* and *Yersinia spp.* (Fernandez-Cassi *et al.* 2020). The next most abundant bacteria, accounting for about a fifth of the reads, were assigned to the Phylum *Firmicutes*: about 50% to the Order *Clostridiales*, 30% to *Lactobacillales* and 20% to *Bacillales*, each of which also includes a number of pathogenic taxa, such as *Clostridium*, *Streptococcus*, *Enterococcus faecium* and *Bacillus cereus*. The next most abundant bacteria, also accounting for about a fifth of the reads, were assigned to the Phylum *Bacteroidetes*: about 40% to the genus *Bacteroides* (including the pathogenic *B. fragilis*), 40% to other *Bacteroidales* and 20% to a variety of *Flavobacteriales*. The remaining fifth of bacterial reads were mostly assigned to the Order Fusobacteriales (primarily *Fusobacterium varium*) in the Phylum *Fusobacteria*, with a minor fraction going to a range of *Actinobacteria*.

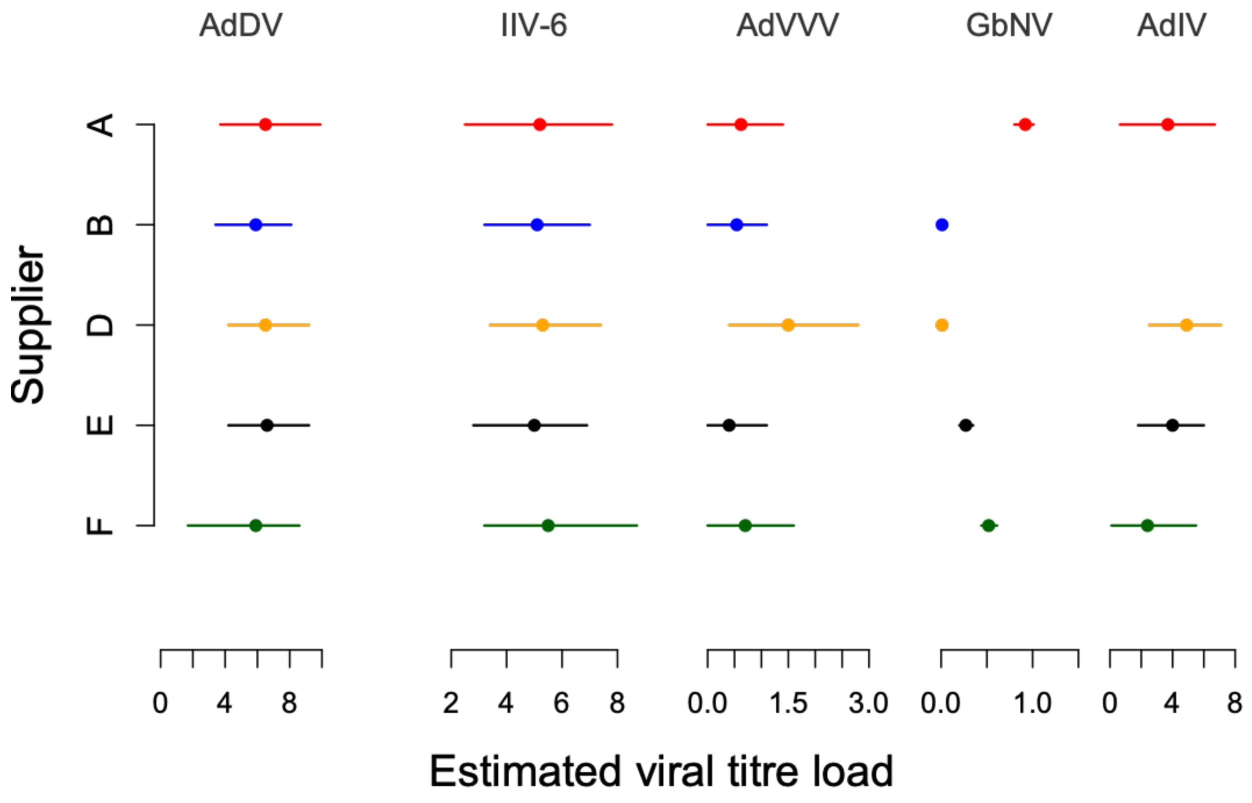

Supplementary Figure 2. Estimated mean titre levels and their associated 95% Confidence Intervals for five viruses (*Acheta domesticus* densovirus – AdDV; Invertebrate Iridescent virus 6 – IIV-6; *Acheta domesticus* volvovirus – AdVVV; *Gryllus bimaculatus* nudivirus – GbNV and *Acheta domesticus* Iflavirus – AdIV) in *Acheta domesticus* samples obtained from five different Swedish retailers. These estimates were generated from intercept-only Bayesian random-effects models (see Supplementary Appendix 1).

SampleID	Supplier	Date	Species	Stage	Sample	Individuals	Condition
AD:0dk	A	2017-05-03	*Acheta domesticus*	adult	frass	n.a.	dried
AD:001	B	2019-07-01	*Acheta domesticus*	juvenile	insect	1	live
AD:002	B	2019-07-01	*Acheta domesticus*	juvenile	insect	1	live
AD:003	B	2019-07-01	*Acheta domesticus*	adult	insect	1	live
AD:004	B	2019-07-01	*Acheta domesticus*	adult	insect	1	live
AD:057	C	2020-01-22	*Gryllus bimaculatus*	adult	insect	3	decomposed
AD:061	C	2020-01-22	*Gryllus bimaculatus*	adult	insect	3	decomposed
AD:065	C	2020-01-22	*Gryllus bimaculatus*	adult	frass	n.a.	dried
AD:069	D	2020-06-28	*Acheta domesticus*	nymph	insect	5	live
AD:072	D	2020-06-29	*Acheta domesticus*	nymph	frass	n.a.	dried
AD:073	D	2020-06-29	*Acheta domesticus*	juvenile	frass	n.a.	dried
AD:074	D	2020-06-29	*Acheta domesticus*	adult	frass	n.a.	dried
AD:075	E	2020-09-09	*Acheta domesticus*	adult	insect	1	live
AD:076	E	2020-09-09	*Acheta domesticus*	adult	insect	1	live
AD:077	E	2020-09-09	*Acheta domesticus*	adult	insect	1	live
AD:078	F	2020-09-25	*Acheta domesticus*	nymph	insect	~50	live

Supplementary Table 1. Details of the samples analysed in this study. Shown are sample ID, an anonymized label for the different suppliers, species of cricket analysed, type of sample analysed (frass, nymph, juvenile or adult insect), number of individuals in each sample and the condition the sample was received in prior to processing.

Virus	Target	Primers	Sequence	Size	Reference
Acheta domesticus densovirus AdDV	Capsid Protein	advp2-f (F)<br>advp-rev (R)	CGTAACCCGGATTATCT<br>GGTCTTGCTACTCTAAATC	305	Szelei *et al.* 2011
Invertebrate iridovirus IIV-6	Major Capsid Protein	IIV-MCP (F)<br>IIV-MCP (R)	TGGTTYACCCAAGTACCKGTTAG<br>ATGCKGACCATTCGCTTC	73	Papp *et al.* 2014
Gryllus bimaculatus nudivirus GbNV	VP91 Capsid Protein	VP91-F (F)<br>VP91-R (R)	CTTCTCACGGTCGAGTTTCTTC<br>CGTCTGCTAAACAAGCTACTCT	291	current study
Acheta domesticus mini Ambidensovirus AdMADV	Capsid Protein	AdMADV (F)<br>AdMADV (R)	CTCGTCCCCACGATGG<br>ATTGTGAAAGTAGGCATTCGTG	388	current study
Acheta domesticus volvovirus AdVVV	Capsid Protein	AdVVV (F)<br>AdVVV (R)	TGGCTGCTTCTCGTTTGG<br>CTTTATCTCAACAGCATCGGG	432	current study
Acheta domesticus iflavirus AdIV	RNA polymerase	AdIF-F (F)<br>AdIF-R (R)	CCATGAGAGACACTTCATCTG<br>CCGTTCATCTTTCAGAAAGAG	212	de Miranda *et al.* 2021
Cricket paralysis virus CrPV	RNA polymerase	CrPV-P1 (F)<br>CrPV-P2 (R)	TCCTCAAGCCATGTGTATAGGA<br>GTGGCTGAAATACTATCTCTGG	585	Kerr *et al.* 2018
Acheta domesticus virus AdV	Capsid Protein	AdV (F)<br>AdV (R)	CAGCCATTCCTAATCCAGG<br>GTGTGCACGCACAATGGG	356	current study
Slow bee paralysis virus SBPV	VP1	SBPV-F3177 (F)<br>SBPV-B3363 (R)	GYGCTTTAGTTCAATTRCC<br>ATTATRGGACGTGARAATATAC	226	de Miranda *et al.* 2010

Supplementary Table 2. Details of the PCR assays used in the study. Shown are the virus name and acronym, the genomic region targeted by the assay, the names and sequences of the primers used, the size (number of base-pairs) of the expected PCR product and the original reference for the assay. The assays for GbNV, AdMADV, AdVVV and AdV were designed as part of the current study.

3. Supplementary Appendix 1

For the first statistical models where we were interested in estimating the viral titres of each supplier, the model structure was a random-effects intercept-only model, where repeated measures from each supplier were used to estimate a different mean (intercept) for each supplier (subscript j) (eq1):

$$\text{Viral titre} \sim \text{Normal}(\text{alpha}_j, \text{sigma})$$
$$\text{alpha}_j \sim \text{Normal}(\text{mu.alpha}, \text{sigma.alpha}) \qquad \text{(eq1)}$$

For the second set of statistical tests where we were interested in correlations between viral titres, we extended this simple model in eq1 to include an explanatory variable (i.e. the viral titre of another virus) to explain the viral load of the first virus (eq2):

$$\text{Viral titre 1} \sim \text{Normal}(\text{mu}, \text{sigma})$$
$$\text{mu} = \text{alpha}_j + \text{beta}*\text{viral titre 2}$$
$$\text{alpha}_j \sim \text{Normal}(\text{mu.alpha}, \text{sigma.alpha}) \qquad \text{(eq2)}$$

Here we examined the parameter estimates for 'beta' which represented the estimated change in viral titre 1 for every unit change in viral titre 2. For these comparisons we used AdDV as viral titre 1 and substituted the other 4 viruses where we had data to look for correlations.

The models were run using JAGS called from R (R Core Team 2019) using the 'rjags' package (Plummer 2018). Models were run for 10000 iterations and trace plots examined for stability. All estimated parameters showed convergence of the trace plots and models showed good fit based on posterior predictive checks.

Supplementary References

Andrews, S. (2010). FastQC: A quality control tool for high throughput sequence data. Babraham Bioinformatics Group. http://www.bioinformatics.babraham.ac.uk/projects/fastqc.

Buchfink, B., Xie, C., Huson, D.H. (2015). Fast and sensitive protein alignment using DIAMOND. Nat Methods. 12, 59-60. doi: 10.1038/nmeth.3176.

Fernandez-Cassi, X., Söderqvist, K., Bakeeva, A., Vaga, M., Dicksved, J., Vagsholm, I.*et al.* (2020). Microbial communities and food safety aspects of crickets (Acheta domesticus) reared under controlled conditions. J. Insects as Food and Feed 6, 429 - 440. DOI: 10.3920/JIFF2019.0048.

Gordon, A., Hannon, G.J. (2010). Fastx-Toolkit: FASTQ/a short-reads pre-processing tools. http://hannonlab.cshl.edu/fastx_toolkit/

Huson, D.H., Beier, S., Flade, I., Górska, A., El-Hadidi, M., Mitra, S. *et al.* (2016). MEGAN Community Edition - Interactive Exploration and Analysis of Large-Scale Microbiome Sequencing Data. PLoS Comput Biol. 12, e1004957. doi: 10.1371/journal.pcbi.1004957.

Kearse, M., Moir, R., Wilson, A., Stones-Havas, S., Cheung, M., Sturrock, S. *et al.* (2012). Geneious Basic: an integrated and extendable desktop software platform for the organization and analysis of sequence data. Bioinformatics 28, 1647-1649. doi: 10.1093/bioinformatics/bts199.

Kerr, C.H., Dalwadi, U., Scott, N.E., Yip, C.K., Foster, L.J., Jan, E. (2018). Transmission of Cricket paralysis virus via exosome-like vesicles during infection of Drosophila cells. Sci Rep. 8, e17353. doi: 10.1038/s41598-018-35717-5.

de Miranda, J.R., Dainat, B., Locke, B., Cordoni, G., Berthoud, H., Gauthier, L., Neumann, P., Budge, G.E., Ball, B.V., Stoltz, D.B. (2010). Genetic characterization of slow bee paralysis virus of the honey bee (*Apis mellifera* L). J Gen Virol. 91, 2524-2530. doi: 10.1099/vir.0.022434-0

de Miranda, J.R., Granberg, F., Onorati, P., Jansson, A., Berggren, Å (2021). Virus prospecting in crickets – Discovery and strain divergence of a novel Iflavirus in wild and cultivated *Acheta domesticus*. Viruses 13, e000. doi: 10.3390/xxxxx

Li, D., Liu, C.M., Luo, R., Sadakane, K., Lam, T.W. (2015). MEGAHIT: an ultra-fast single-node solution for large and complex metagenomics assembly via succinct de Bruijn graph. Bioinformatics 31, 1674-1676. doi: 10.1093/bioinformatics/btv033.

Ondov, B.D., Bergman, N.H., Phillippy, A.M. (2011). Interactive metagenomic visualization in a Web browser. BMC Bioinformatics 12, e385. doi: 10.1186/1471-2105-12-385.

Papp, T., Spann, D., Marschang, R.E. (2014). Development and use of a real-time polymerase chain reaction for the detection of group II invertebrate iridoviruses in pet lizards and prey insects. J Zoo Wildl Med. 45, 219-227. doi: 10.1638/2012-0044.1.

Picard Toolkit (2019). Broad Institute, GitHub Repository: http://broadinstitute.github.io/picard/; Broad Institute.

Plummer, M. (2018). rjags: Bayesian Graphical Models using MCMC. R package version 4-8. URL https://CRAN.R-project.org/package=rjags.

R Core Team (2019). R: A language and environment for statistical computing. R Foundation for Statistical Computing, Vienna, Austria. URL https://www.R-project.org/.

Szelei, J., Woodring, J., Goettel, M.S., Duke, G., Jousset, F-X., Liu, K.Y. *et al.* (2011). Susceptibility of North-American and European crickets to Acheta domesticus densovirus (AdDNV) and associated epizootics. J Inv Pathology 106, 94-399. doi: 10.1016/j.jip.2010.12.009.
